# Effectiveness of two different doses of rituximab for the treatment of rheumatoid arthritis in an international cohort: data from the CERERRA collaboration

**DOI:** 10.1186/s13075-016-0951-z

**Published:** 2016-02-16

**Authors:** Katerina Chatzidionysiou, Elisabeth Lie, Evgeny Nasonov, Galina Lukina, Merete Lund Hetland, Ulrik Tarp, Ioan Ancuta, Karel Pavelka, Dan C. Nordström, Cem Gabay, Helene Canhão, Matija Tomsic, Piet L. C. M. van Riel, Juan Gomez-Reino, Tore K. Kvien, Ronald F. van Vollenhoven, on behalf of the Rheumatic Diseases Portuguese Register

**Affiliations:** Department of Medicine, Karolinska Institute, Unit for Clinical Research Therapy, Inflammatory Diseases (ClinTrid), D1:00, Karolinska Universitetssjukhustet, 171 76, Stockholm, Sweden; Department of Rheumatology, Diakonhjemmet Hospital, Oslo, Norway; ARBITER, Institute of Rheumatology, Moscow, Russia; DANBIO, Department of Rheumatology, Copenhagen University Hospital at Glostrup, Copenhagen, Denmark; Department of Rheumatology, Aarhus University Hospital, Aarhus, Denmark; Cantacuzino Hospital, Bucharest, Romania; Charles University, Prague, Czech Republic; ROB-FIN Helsinki University Central Hospital, Helsinki, Finland; SCQM registry, University Hospital of Geneva, Geneva, Switzerland; Rheumatology Research Unit, Instituto de Medicina Molecular, Lisbon, Portugal; University Medical Center, Ljubljana, Slovenia; Radboud University Nijmegen Medical Centre, Nijmegen, Netherlands; Hospital Clinico Universitet De Santiago, Santiago, Spain

**Keywords:** Rituximab, Rheumatoid arthritis, Observational

## Abstract

**Background:**

The approved dose of rituximab (RTX) in rheumatoid arthritis is 1000 mg × 2, but some data have suggested similar clinical efficacy with 500 mg × 2. The purpose of this study was to compare the effectiveness of the regular and low doses given as first treatment course.

**Methods:**

Twelve European registries participating in the CERERRA collaboration (The European Collaborative Registries for the Evaluation of Rituximab in Rheumatoid Arthritis) submitted anonymized datasets with demographic, efficacy and treatment data for patients who had started RTX. Treatment effectiveness was assessed by DAS28 reductions and EULAR responses after 6 months.

**Results:**

Data on RTX dose were available for 2,873 patients, of whom 2,625 (91.4 %) and 248 (8.6 %) received 1000 mg × 2 and 500 mg × 2, respectively. Patients treated with 500 mg × 2 were significantly older, had longer disease duration, higher number of prior DMARDs, but lower number of prior biologics and lower baseline DAS28 than those treated with 1000 mg × 2. Fewer patients in the low-dose group received concomitant DMARDs but more frequently received concomitant corticosteroids.

Both doses led to significant clinical improvements at 6 months. DAS28 reductions at 6 months were comparable in the 2 dose regimens [mean DeltaDAS28 ± SD -2.0 ± 1.3 (high dose) vs. -1.7 ± 1.4 (low dose), p = 0.23 adjusted for baseline differences]. Similar percentages of patients achieved EULAR good response in the two dose groups, 18.4 % vs. 17.3 %, respectively (p = 0.36).

**Conclusions:**

In this large observational cohort initial treatment with RTX at 500 mg × 2 and 1000 mg × 2 led to comparable clinical outcomes at 6 months.

## Background

Rituximab (MabThera, Rituxan) is a chimeric, monoclonal anti-CD20 antibody approved for the treatment of rheumatoid arthritis (RA) in combination with methotrexate in patients with active RA who have not responded to at least one tumor necrosis factor (TNF) inhibitor. The efficacy and acceptable safety profile of rituximab (RTX) have been demonstrated in randomized controlled trials [1, 2] and in large observational cohorts [3, 4]. The approved dose is 1000 mg × 2 (with a 2-week interval) per treatment course.

There is, however, evidence suggesting that a lower dose of RTX, 500 mg × 2, is also effective, although not approved. In the SERENE trial both 500 mg × 2 and 1000 mg × 2 of RTX significantly improved clinical outcomes (based on criteria of the American College of Rheumatology (ACR) responses, the European League Against Rheumatism (EULAR) responses, and improvement in the Disease Activity Score in 28 joints (DAS28) and in the Health Assessment Questionnaire (HAQ)) compared to placebo in a biologic-agent-naïve population of patients with RA [5]. The MIRROR, DANCER and IMAGE trials yielded similar results [6–8]. In all the above trials no significant difference was detected between the different doses in almost all clinical outcomes.

The purpose of this study was to assess and compare the effectiveness at 6 months of the higher (1000 mg × 2) and lower (500 mg × 2) dose of RTX given as the first treatment course in a merged dataset from observational cohorts.

## Methods

The European Collaborative Registries for the Evaluation of Rituximab in Rheumatoid Arthritis (CERERRA) is an investigator-led initiative aiming to evaluate clinical aspects of RTX use in patients with RA [4, 9]. Twelve participating European registries (from the Czech Republic, Denmark, Finland, The Netherlands, Norway, Portugal, Romania, Russia, Slovenia, Spain, Sweden and Switzerland) submitted fully anonymized datasets with baseline demographic and disease characteristics, including age, gender, disease duration, number of previous synthetic and biological disease-modifying anti-rheumatic drugs (DMARDs), rheumatoid factor (RF) and anti-cyclic citrullinated peptide antibody (anti-CCP) status of all patients with an established diagnosis of RA who started treatment with RTX. Data are collected prospectively in each register. Ethical approval for the use of register data from each register was obtained by local authorities of each country. The Regional Ethical Review Board in Stockholm approved the collection and analysis of anonymized data from the twelve participating registers. Informed consent was obtained from each patient before inclusion in each register, according to local regulations. Disease activity markers at baseline and after 3 and 6 months were also provided (number of swollen and tender joints, visual analog scales (VAS) for pain, patient’s and physician’s global assessment, DAS28 and erythrocyte sedimentation rate (DAS28-ESR), and HAQ). Information about RA treatment, such as doses of RTX and use of concomitant DMARDs and glucocorticoids was also included in the dataset. Effectiveness of RTX in the higher (1000 mg × 2) and the lower dose (500 mg × 2) was assessed by DAS28 and HAQ status at 3 and 6 months, by the improvement of DAS28 and HAQ at 3 and 6 months, by disease activity at 3 and 6 months based on DAS28 status and by EULAR responses at 6 months. A small number of patients were treated with other than the above doses (e.g., 750 mg) or did not provide information on the RTX dose and were therefore not included in the analysis.

### Statistical analysis

Baseline characteristics of the two groups were analyzed by means of descriptive statistics. For normally distributed variables mean ± standard deviation (SD) and the independent samples *t* test was used, and median (interquartile range (IQR)) and the Mann–Whitney *U* test were used for the non-normally distributed variables. The chi-square test was used for comparison of categorical data.

Changes in DAS28 and HAQ were first compared in unadjusted analysis using the independent samples *t* test. Comparative adjusted analysis with correction for baseline group differences was subsequently performed by analysis of covariance (ANCOVA). In the ANCOVA we adjusted for baseline variables found to differ significantly between the two groups (age, disease duration, number of prior biologic agents used, baseline DAS28, and concomitant use of DMARDs) and for those thought to be clinically significant (concomitant use of corticosteroids). The number of prior DMARDs used was not included in the ANCOVA even though it was significantly different between groups, because of the small number of patients with available information in the 500-mg group and because of the high correlation with the number of prior biologic agents. Multivariate logistic regression analysis was performed with EULAR response as the dependent variable (first, analysis of good vs. moderate/no EULAR response and second, analysis of good/moderate vs. no EULAR response) and RTX dose (500 vs. 1000 mg), and several baseline variables as explanatory variables (age, gender, RA disease duration, use of previous biologic agents, baseline DAS28, anti-CCP status, and concomitant use of DMARDs and corticosteroids). Country was included in the model in an additional analysis. All statistical tests were evaluated at the 0.05 significance level. *P* values and 95 % confidence intervals are presented. The statistical analysis was performed with IBM SPSS Statistics version 20.

## Results

The total number of patients included in the cohort was 3,266, and 2,873 patients (88 %) were eligible for analysis. The large majority of patients ([n = 2,625, 91.4 %) received 1000 mg × 2 (higher dose), and 248 patients (8.6 %) received 500 mg × 2 (lower dose). The demographics and baseline disease characteristics for the two treatment groups are shown in Table 1. Patients who were treated with the lower RTX dose were older, had longer disease duration, prior use of fewer biologic agents but more DMARDs, and lower baseline DAS28 than those treated with the higher dose. Additionally, fewer patients in the low-dose group received concomitant DMARDs but more frequently received concomitant corticosteroids. Baseline characteristics for those patients with available DAS28-ESR at 6 months are also shown in Table 1. No significant differences between the two populations (all patients at baseline and patients with available response data at 6 months) were observed, so missingness of data was not informative.

**Table 1:** Baseline demographics, disease and treatment characteristics of patients treated with rituximab 500 mg × 2 or 1000 mg × 2 for all patients in the cohort at baseline and for those with available response data (DAS28-ESR) at 6 months

	All patients			Patients with available response data at 6 months*		
	RTX 500 mg × 2 n = 248	RTX 1000 mg × 2 n = 2625	*P* value (*t* test, chi-square test)	RTX 500 mg × 2 n = 109	RTX 1000 mg × 2 n = 1385	*P* value (*t* test, chi-square test)
Sex,% female	83.9 % (248)	80.3 % (2,625)	0.17	88.1 % (109)	81.2 % (1,125)	0.08
Age, years	55.2 ± 15.8 (247)	52.6 ± 12.6 (2,615)	0.002	55.5 ± 15.1 (109)	51.1 ± 11.8 (1,380)	<0.0001
Disease duration, years	10.5 (5-18) (240)	8.1 (5-14) (2,360)	0.02	10 (5-16.8) (108)	7.5 (5.1-12) (1,329)	0.02
RF, % positive	81.7 % (241)	81.2 % (2,031)	0.84	83 % (106)	81.5 % (876)	0.7
Anti-CCP, % positive	71.3 % (101)	73.4 % (806)	0.64	68 % (50)	74.9 % (363)	0.29
Number of prior biologic agents	0 (0-1) (207)	1 (0-2) (2,560)	<0.0001	0 (0-1) (102)	1 (0-1) (1,371)	0.003
Anti-TNF-naive (%)	58 % (207)	37.5 % (2,560)	<0.0001	58.8 % (102)	37.1 % (1,371)	<0.0001
Number of prior DMARDs	2.6 ± 1.3 (126)	2.4 ± 1.4 (2,248)	0.04	2.7 ± 1.3 (55)	2.3 ± 1.1 (1,256)	0.02
Baseline DAS28-ESR	5.7 ± 1.3 (215)	6.1 ± 1.3 (2,069)	<0.0001	5.9 ± 1.3 (100)	6.3 ± 1.2 (1,344)	0.002
Baseline HAQ score	1.6 ± 0.7 (212)	1.7 ± 0.7 (1,584)	0.48	1.6 ± 0.7 (100)	1.8 ± 0.7 (695)	0.001
Concomitant medication:						
– Any DMARD	72.6 % (248)	83.1 % (2,625)	<0.0001	75.2 % (109)	87.5 % (1,385)	<0.0001
MTX	46.4 % (248)	63.4 % (2,625)	<0.0001	50.5 % (109)	75.1 % (1,385)	<0.0001
Glucocorticoids	65.7 % (248)	59.3 % (2,221)	0.06	71.6 % (109)	64.7 % (983)	0.15

The number of patients with available information for each variable is included in brackets. *RTX* rituximab, *RF* rheumatoid factor, *anti-CCP* anti-cyclic citrullinated peptide antibodies, *DMARDs* disease modifying anti-rheumatic drugs, *DAS28-ESR* Disease Activity Score based on 28 joints and erythrocyte sedimentation rate, *HAQ* health assessment questionnaire, *MTX* methotrexate

**Fig. 1:**
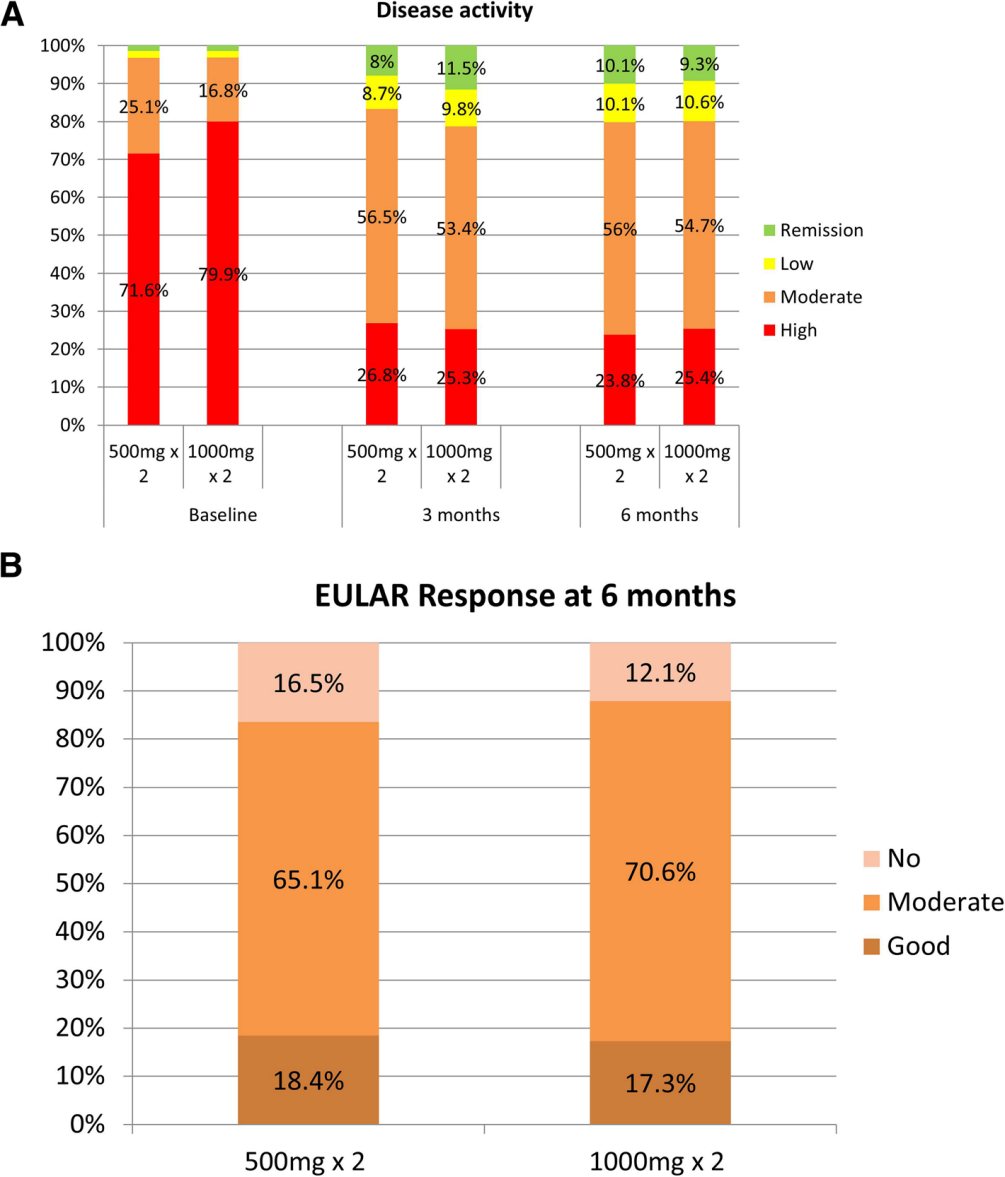
**a** Disease activity based on Disease Activity Score in 28 joints (DAS28)-erythrocyte sedimentation rate (ESR) at baseline, 3 and 6 months in the two treatment groups (rituximab (RTX) 500 mg × 2 and RTX 1000 mg × 2). No significant differences were observed: remission, DAS28 <2.6; low disease activity, 2.6≤ DAS28 ≤3.2; moderate disease activity, 3.2< DAS28 ≤5.1; high disease activity, DAS28 >5.1. **b**. Good, moderate or no European League Against Rheumatism (*EULAR*) response at 6 months for the two treatment groups (RTX 500 mg × 2 and RTX 1000 mg × 2). No significant differences were observed

In the unadjusted analysis, the mean DAS28 improvement at 3 months was greater for patients treated with the higher dose than for those treated with the lower dose (1.9 ± 1.4 (n = 991) vs. 1.3 ± 1.3 (n = 125), *p* <0.0001) and it remained significant in the ANCOVA (*p* = 0.004) (Table 2). The difference in mean DAS28 improvement was also significant at 6 months (2.0 ± 1.3 (n = 1344) vs. 1.7 ± 1.4 (n = 100), *p* = 0.02) in the unadjusted analysis. However, there was no significant difference after adjustment (*p* = 0.23). Inclusion of country as a random variable in the ANCOVA did not change the results. Improvements in function as assessed by the HAQ were also similar between the groups both at 3 and 6 months (Table 2). The proportion of patients with high, moderate and low disease activity and remission based on the DAS28 was similar in the two groups at baseline, 3 and 6 months (Fig. 1a), as was the proportion of EULAR good responders, moderate responders and non-responders at 6 months (Fig. 1b).

**Table 2:** Effectiveness of treatment across the two treatment groups as assessed by DAS28 and HAQ status and changes at 3 and 6 months

	RTX 500 mg × 2	RTX 1000 mg × 2	Unadjusted *p* values (ANOVA)	Adjusted *p* values**
DAS28 baseline	5.7 ± 1.3 (215)	6.1 ± 1.3 (2069)	<0.0001	
DAS28 3 m	4.4 ± 1.2 (138)	4.2 ± 1.3 (1046)	0.15	
DAS28 6 m	4.3 ± 1.3 (109)	4.3 ± 1.2 (1385)	0.99	
DeltaDAS28 3 m	–1.3 ± 1.3 (125)	–1.9 ± 1.4 (991)	<0.0001	0.004
DeltaDAS28 6 m	–1.7 ± 1.4 (100)	–2.0 ± 1.3 (1344)	0.02	0.23
HAQ baseline	1.6 ± 0.7 (212)	1.6 ± 0.7 (1584)	0.48	
HAQ 3 m	1.3 ± 0.7 (127)	1.3 ± 0.7 (957)	0.83	
HAQ 6 m	1.2 ± 0.7 (109)	1.3 ± 0.7 (912)	0.21	
DeltaHAQ 3 m	–0.3 ± 0.5 (115)	–0.5 ± 0.6 (859)	0.02	0.10
DeltaHAQ 6 m	-0.4 ± 0.6 (103)	–0.5 ± 0.7 (826)	0.13	0.27

Crude and adjusted *p* valued are presented. **Analysis of covariance (ANCOVA) adjusted for age, sex, disease duration, number of prior biologic agents used, baseline Disease Activity Score in 28 joints (DAS28), concomitant use of disease-modifying anti-rheumatic drugs and glucocorticoids. *RTX* rituximab, *HAQ* Health Assessment Questionnaire, *m* months

Multivariate logistic regression analysis was performed to examine the possible association between RTX dose and good (vs. moderate/no) and good/moderate (vs. no) EULAR response, with adjustment for possible confounders, such as age, gender, RA disease duration, previous use of biologic agents, baseline DAS28, anti-CCP status, and use of concomitant DMARDs and corticosteroids. RTX dose (lower dose vs. higher dose) was not a statistically significant predictor of achieving a good EULAR response (odds ratio (OR) 1.08, 95 % CI 0.40, 2.94, *p* = 0.88) or good/moderate response EULAR (OR 1.22, 95 % CI 0.37, 4.09, *p* = 0.74). Similar results were observed when country was introduced into the model.

## Discussion

In this study based on data from the CERERRA collaboration RTX provided significant clinical improvement at 3 and 6 months in patients with active RA. On comparison of the higher (1000 mg × 2) and lower dose (500 mg × 2) of RTX there was a significant difference in DAS28 improvement at 3 months but not in clinical effectiveness, as assessed by change in DAS28 and HAQ score at 6 months, after adjusting for baseline characteristics. EULAR response rates and remission rates were also similar between groups. The results of our study are thus consistent with those from the SERENE, IMAGE and MIRROR trials [5, 6, 8]. In the MIRROR trial the percentage of patients with good/moderate EULAR responses was borderline significantly higher in the 1000 mg × 2 (89 %) compared to the 500 mg × 2 group (73 %) (*p* = 0.05). The overall conclusions of the MIRROR trial was that the two RTX doses could not be clearly differentiated, although some clinical outcomes were in favor of the higher dose [6]. A recent systematic review and meta-analysis made a similar conclusion [10]. The similar effectiveness of lower dose RTX in patients with RA in clinical practice might have important pharmaco-economic implications for health systems globally.

Hitherto there have been no proper dose-response studies for RTX in RA. What is considered the “autoimmune dose and protocol” was developed by Professor Jo Edwards based on a small number of patients [11]. Our study provides some additional evidence about the lack of any striking difference and perhaps no clinically significant difference between the two different doses of RTX used in clinical practice. Recently interesting data from the UK Leeds group showed that response to RTX may be more dependent on the B-cell depletion rather than the dose. Although in the small number of patients studied “incomplete” peripheral blood depletion was more often seen in the patients treated with the lower dose, “complete” depletion was seen in both groups and correlated better with response than dose itself [12]. The depletion of B cells with anti-CD20 treatment varies between individuals, even with the same dose, as shown in several animal and human studies, but tends to be consistent in the same individuals [13–15]. This suggests that individual factors are important in determining the final extent of depletion.

There are several limitations that should be addressed: the observational character of the study, the different size of the two treatment groups under comparison (only 248 patients treated with the lower dose), and the fact that the two groups compared were not balanced for all baseline characteristics. Hence, there is a risk of channeling bias, as patients treated with the lower dose were older, had longer disease duration, lower disease activity at baseline and less prior use of biologic agents, and were more often treated with corticosteroids and less often with concomitant DMARDs. The lower-dose group may represent a population of patients with more comorbidities, for whom the treating rheumatologist chose the lower dose of RTX. However, such a population would be more prone to have a worse response to therapy, and therefore confounding by indication would bias the results against the lower dose of RTX. On assessing the potential influence of corticosteroids on response, the percentage of patients with concomitant use of corticosteroids in the two groups was quite similar and not statistically significant (Table 1). Additionally, in the ANCOVA we adjusted for concomitant use of corticosteroids (Table 2) as it is clinically significant.

The lack of radiological data is an additional limitation of the study. The *golden triad* of current treatment guidelines in RA is remission (or low disease activity when remission is not possible), preservation of functional ability, and prevention of structural damage. Tak et al. showed in the IMAGE study that the 1000 mg × 2 RTX, but not the 500 mg × 2 dose, significantly inhibited progression of joint damage during the first 6 months, but inhibition of structural progression was similar from 6 months onwards [8, 16]. However, the IMAGE trial included MTX-naïve patients of whom the majority had early RA, and its population was thus different from the population in our study. It would be interesting to further evaluate the ability of the lower RTX dose to prevent radiological progression in an established RA population that is more consistent with the routine use of RTX. The length of sustained response was not examined in the present study. The risk that the lower dose might be associated with shorter response cannot be ruled out and should be assessed in future studies. The large number of patients included in the cohort, which made the comparison of the different doses of RTX possible, and the real-life character of the study are important strengths of the study.

## Conclusions

In this large observational cohort initial treatment with RTX at 500 mg × 2 and 1000 mg × 2 led to comparable clinical outcomes after 6 months. This result may have some important cost implications in the treatment of patients with RA.

## Abbreviations

ACR: American College of Rheumatology
ANCOVA: analysis of covariance
Anti-CCP: anti-cyclic citrullinated peptide
CERERRA: European Collaborative Registries for the Evaluation of Rituximab in Rheumatoid Arthritis
CRP: C-reactive protein
DAS: Disease Activity Score
DMARDs: disease modifying anti-rheumatic drugs
ESR: erythrocyte sedimentation rate
EULAR: European League Against Rheumatism
HAQ: health assessment questionnaire
MTX: methotrexate
OR: odds ratio
RA: rheumatoid arthritis
RF: rheumatoid factor
RTX: rituximab
TNF: tumor necrosis factor
