# Erratum to: Effectiveness of two different doses of rituximab for the treatment of rheumatoid arthritis in an international cohort: data from the CERERRA collaboration

**DOI:** 10.1186/s13075-016-1048-4

**Published:** 2016-06-20

**Authors:** Katerina Chatzidionysiou, Elisabeth Lie, Evgeny Nasonov, Galina Lukina, Merete Lund Hetland, Ulrik Tarp, Ioan Ancuta, Karel Pavelka, Dan C. Nordström, Cem Gabay, Helene Canhão, Matija Tomsic, Piet L. C. M. van Riel, Juan Gomez-Reino, Tore K. Kvien, Ronald F. van Vollenhoven

**Affiliations:** Department of Medicine, Karolinska Institute, Unit for Clinical Research Therapy, Inflammatory Diseases (ClinTrid), D1:00, Karolinska Universitetssjukhustet, Stockholm, 171 76 Sweden; Department of Rheumatology, Diakonhjemmet Hospital, Oslo, Norway; ARBITER, Institute of Rheumatology, Moscow, Russia; DANBIO, Department of Rheumatology, Copenhagen University Hospital at Glostrup, Copenhagen, Denmark; Department of Rheumatology, Aarhus University Hospital, Aarhus, Denmark; Cantacuzino Hospital, Bucharest, Romania; Charles University, Prague, Czech Republic; ROB-FIN Helsinki University Central Hospital, Helsinki, Finland; SCQM registry, University Hospital of Geneva, Geneva, Switzerland; Rheumatic Diseases Portuguese Register, Rheumatology Research Unit, Instituto de Medicina Molecular, Lisbon, Portugal; University Medical Center, Ljubljana, Slovenia; Radboud University Nijmegen Medical Centre, Nijmegen, Netherlands; Hospital Clinico Universitet De Santiago, Santiago, Spain

## Erratum

Unfortunately, after publication of this article [1] it was noticed that the author list was incorrectly captured during the production process. The original author list contained the group “Rheumatic Diseases Portuguese Register” however this was included erroneously and instead should only be listed as the affiliation for Helene Canhão. The correct author list can be seen above and the original article has been updated to reflect this change.
